# Morphological updates and molecular description of *Heterosentis holospinus* Amin, Heckmann, & Ha, 2011 (Acanthocephala, Arhythmacanthidae) in the Pacific Ocean off Vietnam

**DOI:** 10.1051/parasite/2019072

**Published:** 2019-12-19

**Authors:** Omar M. Amin, Sara M. Rodríguez, Richard A. Heckmann

**Affiliations:** 1 Institute of Parasitic Diseases 11445 E. Via Linda 2-419 85259 Scottsdale AZ USA; 2 Instituto de Ciencias Marinas y Limnológicas, Facultad de Ciencias, Universidad Austral de Chile Campus Isla Teja s/n Valdivia Chile; 3 Department of Biology, Brigham Young University 1114 MLBM 84602 Provo UT USA

**Keywords:** Acanthocephala, *Heterosentis holospinus*, Vietnam, New features, Molecular profile

## Abstract

*Heterosentis holospinus* Amin, Heckmann & Ha, 2011 (Arhythmacanthidae) was first described from the striped eel catfish, *Plotosus lineatus* (Plotosidae) in Halong Bay, Vietnam. New morphological information, scanning electron microscope images, molecular analysis, and Energy Dispersive X-ray analysis (EDXA) of hooks of specimens of *H. holospinus* from a new collection from the common ponyfish, *Leiognathus equulus* (Leiognathidae), in Quang Binh, Gulf of Tonkin, Vietnam are reported here for the first time. Additional details of the anterior trunk cone, proboscis hooks, wholly spined trunk, duck-bill-like spines with micropores, and micropore distribution, are described. The unique metal composition of hooks (EDXA) demonstrated a considerably higher level of calcium and phosphorus but lower level of sulfur at the hook basal arch than at the hook tip and edge. An analysis of our new sequences of cytochrome oxidase 1 (COI) showed that *H. holospinus* had low genetic variation and two haplotypes.

## Abstract (fr)

*Heterosentis holospinus* Amin, Heckmann & Ha, 2011 (Arhythmacanthidae) a été décrit pour la première fois chez le poisson-chat *Plotosus lineatus* (Plotosidae) dans la baie d’Halong, au Vietnam. Des nouvelles informations morphologiques, des images au microscope électronique à balayage, une analyse moléculaire et une analyse par rayons X à dispersion d’énergie (EDXA) des crochets de spécimens d’*H. holospinus* provenant d’une nouvelle récolte de *Leiognathus equulus* (Leiognathidae), à Quang Binh, golfe du Tonkin, Vietnam sont données pour la première fois. Des détails supplémentaires sur le cône antérieur du tronc, les crochets du proboscis, le tronc entièrement épineux, les épines en forme de bec de canard avec des micropores et la distribution des micropores sont décrits. La composition métallique unique des crochets (EDXA) a démontré une teneur en calcium et en phosphore considérablement plus élevée, mais une teneur en soufre plus faible au niveau de l’arcade basale du crochet qu’à la pointe et au bord du crochet. Une analyse de nos nouvelles séquences de cytochrome oxydase 1 (COI) a montré qu’*H. holospinus* présentait une faible variation génétique et deux haplotypes.

## Introduction

Most of the recent taxonomic work on the Acanthocephala from Vietnam has been reported by the Amin–Heckmann–Ha team since 2000. We have described over 50 acanthocephalan species and higher taxa from freshwater and marine fishes, amphibians, reptiles, birds, and mammals in Vietnam. A complete listing of this taxonomic literature can be found in Amin et al. [7]. Three other species of *Rhadinorhynchus* and one species of *Gorgorhynchus* were previously reported from marine fishes in Vietnam [9].

Eighteen species of acanthocephalans in five families were more recently collected from fishes in the Pacific and amphibians in central Vietnam in 2016 and 2017. Amin et al. [3] discussed the taxonomic status and history of the genus *Heterosentis* Van Cleave, 1931, with *Arhythmacanthus* Yamaguti, 1935 as its junior synonym, based on the number of different types of hooks on the proboscis, listed its 15 known species and their distribution, and noted that “the distribution of species of *Heterosentis* corresponds to that of its host species from the Indo-West Pacific into the Eastern Mediterranean via the Red Sea.”

In the present report, we expand the morphological description of *Heterosentis holospinus* Amin, Heckmann, Ha, 2011 (Arhythmacanthidae) from the common ponyfish, *Leiognathus equulus* (Forsskål) (Leiognathidae) in Halong Bay, Vietnam. The common pony fish is distributed in brackish and marine waters from East Africa to Fiji, in the Indian and Western Pacific oceans, Red Sea, and the Arabian Gulf [16]. We also provide, for the first time, descriptions of its molecular profile and Energy Dispersive X-ray pattern, as well as its micropores.

## Materials and methods

### Collections

Specimens of *H. holospinus* were collected from an undetermined number of individuals of the common ponyfish, *Leiognathus equulus* (Forsskål) (Leiognathidae), which were concurrently infected with other acanthocephalans, off the central Pacific coast of Vietnam at Quang Binh (17°30′ N 106°20′ E) in the Gulf of Tonkin near Halong Bay on May 15, 2017, and previously recorded but not described [22] from the same host species in Quang Ninh, also in the Halong Bay area.

Freshly collected specimens were extended in water until proboscides everted then fixed in 70% ethanol for transport to our Arizona, USA laboratory for processing and further studies.

### Methods

Worms were punctured with a fine needle and subsequently stained in Mayer’s acid carmine, destained in 4% hydrochloric acid in 70% ethanol, dehydrated in ascending concentrations of ethanol (24 h each), and cleared in 100% xylene then in 50% Canada balsam, and 50% xylene (24 h each). Whole worms were then mounted in Canada balsam. Measurements are in micrometers, unless otherwise noted; the range is followed by the mean values between parentheses. Width measurements represent maximum width. Trunk length does not include proboscis, neck, or bursa.

Specimens were deposited in the University of Nebraska’s State Museum’s Harold W. Manter Laboratory (HWML) collection no. 139404 (voucher specimens on 1 slide), Lincoln, Nebraska, USA.

### SEM (scanning electron microscopy)

Two specimens that had been fixed and stored in 70% ethanol were processed for SEM following standard methods [27]. These included critical point drying (CPD) in sample baskets and mounting on SEM sample mounts (stubs) using conductive double-sided carbon tape. Samples were coated with gold and palladium for 3 min using a Polaron #3500 sputter coater (Quorum (Q150 TES) https://www.quorumtech.com), establishing an approximate thickness of 20 nm. Samples were placed and observed in an FEI Helios Dual Beam Nanolab 600 (FEI, Hillsboro, Oregon, USA) Scanning Electron Microscope with digital images obtained in the Nanolab software system (FEI) and then transferred to a USB for future reference. Samples were received under low vacuum conditions using 10 kV, spot size 2, 0.7 Torr using a GSE detector.

### Energy dispersive X-ray analysis

Standard methods were used for preparation similar to the SEM procedure. Specimens were examined and positioned with the above SEM instrument, which was equipped with a Phoenix energy-dispersive X-ray analyzer (FEI). X-ray spot analysis and live scan analysis were performed at 16 kV with a spot size of five and results were recorded on charts and stored with digital imaging software attached to a computer. The TEAM (Texture and Elemental Analytical Microscopy) software system (FEI) was used. Data were stored on a USB for future analysis. The data included weight percent and atom percent of the detected elements following correction factors.

### Ion sectioning of hooks

A dual-beam SEM with a gallium (Ga) ion source (GIS) is used for the LIMS (Liquid Ion Metal Source) part of the process. The hooks of the acanthocephalans were centered on the SEM stage and cross sectioned using a probe current between 0.2 nA and 2.1 nA, according to the rate at which the area is cut. The time of cutting was based on the nature and sensitivity of the tissue. Following the initial cut, the sample also underwent a milling process to obtain a smooth surface. The cut was then analyzed with X-ray at the tip, middle, and base of hooks for chemical ions with an electron beam (Tungsten) to obtain an X-ray spectrum. Results were stored with the attached imaging software. The intensity of the GIS was variable, according to the nature of the material being cut.

### Molecular methods

Genetic comparisons and phylogenetic analyses were based on a fragment of 621 bp of the mitochondrial cytochrome oxidase I (COI) gene. Sequences of four individuals of *Heterosentis holospinus* from ponyfish *Leiognathus equulus* from two collections constituted the Vietnam sample. The samples were digested overnight at 55 °C and genomic DNA was isolated using a commercial extraction kit (Wizard^®^ Genomic DNA Purification Kit, Promega, Madison, WI, USA) and the COI gene was amplified using the primers detailed by Folmer et al. [15]. PCR amplification of COI was carried out in 25 μL. Thermal cycling parameters for rDNA amplifications included a denaturation period of 3 min at 94 °C, followed by denaturation of 30 s, annealing of 45 s at 48 °C, and extension of 1 min at 72 °C, with final incubation at 72 °C for 10 min [37]. Amplicons were sequenced using an external sequencing service (Macrogen Inc., Seoul, South Korea). New DNA sequences were edited using Codon-Code (Codon Code Aligner, Dedham, Massachusetts, USA) and deposited in GenBank (MN715352–MN715355). The 4 new sequences were integrated to a matrix with other sequences downloaded from GenBank. Sampling included all available sequences belonging to species of the family Arhythmacanthidae along with sequences of the genera *Acanthocephalus* (Echinorhynchidae) and *Filisoma* (Cavisomidae), which are phylogenetically close to Arhythmacanthidae. In addition, sequences of one species of each family of the orders Echinorhynchida and Polymorphida were also included (Table 1). As such, the ingroup encompasses 31 sequences; sequences of *Atactorhynchus duranguensis*, *Hebesoma violentum* and *Mayarhynchus karlae* which belong to the Class Eoacanthocephala were used as the outgroup.

**Table 1:** Species of acanthocephalans, their hosts, origins, and GenBank accession numbers used for phylogenetic analysis based on the *cox*1 gene.

Order (family)	Species	Host	Location	References	Genbank access
Echinorhynchida (Arhythmacanthidae)	*Heterosentis holospinus* (4)*	*Leiognathus equulus*	Gulf of Tonkin, Vietnam	This study	MN715352–MN715355
	*Acanthocephaloides Propinquus* (1)	*Gobius bucchichii*	Unknown	Garcia-Varela and Nadler [17]	DQ089713
Echinorhynchida (Cavisomidae)	*Filisoma caudate* (1)	*Kyphosus incisor*	Rio de Janeiro Brazil	Costa-Fernandes et al. [14]	MH004408
	*Filisoma bucerium* (1)	*Kyphosus elegans*	Unknown	Garcia-Varela and Nadler [18]	DQ089722
	*Neorhadinorhnchus nudus* (1)	*Auxis thazard*	South Sea, China	Li et al. [28, 29]	MG838935
Echinorhynchida (Echinorhynchidae)	*Pseudoacanthocephalus lucidus* (1)	*Rana ornativentris*	Hokkaido, Japan	Nakao [32]	LC100064
	*Acanthocephalus lucii* (1)	*Perca fluviatilis*	Lake Bleasby, United Kingdom	Wayland et al. [42]	KP261016
	*Acanthocephalus anguillae* (1)	*Perca fluviatilis*	Austria	Benesh et al. [11]	AM039865
	*Acanthocephalus nanus* (1)	*Cynops pyrrhogaster*	Uozu, Japan	Nakao [32]	LC100070
	*Acanthocephalus dirus* (1)	*Asellus aquaticus*	Unknown	Garcia-Varela and Nadler [18]	DQ089718
	*Acanthocephalus clavula* (1)	*Perca fluviatilis*	Ireland	Benesh et al. [11]	AM039866
	*Echinorhynchus bothniensis* (1)	*Osmerus eperlanus*	Lake Keitele, Finland	Wayland et al [42]	KP261018
Echinorhynchida (Gymnorhadinorhynchidae)	*Gymnorhadinorhynchus* sp. (1)	*Regalecus russelii*	Japan	Steinauer et al. [39]	MK012667
Echinorhynchida (Illiosentidae)	*Dentitruncus truttae* (1)	*Salmo trutta*	Krka river, Croatia	Irena et al. [24]	JX460902
	*Dollfusentis chandleri* (1)	Unknown croaker	Unknown	Baker and Sotka, [10]	DQ320486
	*Koronacantha mexicana* (1)	*Pomadasys leuciscus*	Unknown	Garcia-Varela and Nadler [18]	DQ089708
	*Koronacantha pectinaria* (1)	*Microlepidotus brevipinnis*	Unknown	Garcia-Varela and Nadler [18]	DQ089707
Polymophida (Centrorhynchidae)	*Sphaerirostris lanceoides* (1)	*Bufo gargarizans* Cantor	Yuyao County, China	Kang and Li [25]	MG931943
Polymorphida (Rhadinorhynchidae)	*Gorgorhynchoides bullocki* (1)	*Eugerres plumiere*	Unknown	Garcia-Varela and Nadler [18]	DQ089715
Polymorphida (Polymorphidae)	*Profilicollis altmani* (1)	*Leucophaeus modestus*	Curiñanco beach, Valdivia, Chile	Rodríguez et al. [38]	KX702245
Polymorphida (Transvenidae)	*Transvena annulospinosa* (1)	*Anampses neoguinaicus*	Unknown	García-Varela and Nadler [18]	DQ089711
Polymorphida (Plagiorhynchidae)	*Plagiorhynchus cylindraceus* (1)	Unknown	Unknown	García-Varela and Nadler [18]	DQ089714
Polymorphida (Pomporhynchidae)	*Longicollum pagrosomi* (1)	*Oplegnathus fasciatus*	Zhoushan Islands, China	Li et al. [28, 29]	KY490048
	*Pomporhynchus bulbocolli* (1)	*Moxostoma erythrurum*	Wolf river, USA	Garcia-Varela et al. [20]	KY911323
Neoechinorhynchida (Neoechinorhynchidae)	*Atactorhynchus duranguensis* (1)	*Cyprinodon meeki*	South-eastern, Mexico	Pinacho-Pinacho et al. [35]	KY077097
Neoechinorhynchida (Neoechinorhynchidae)	*Hebesoma violentum* (1)	*Perccottus glenii*	North-east Asia, Russia	Malyarchuk et al. [31]	KF156893
Neoechinorhynchida (Neoechinorhynchidae)	*Mayarhynchus karlae* (1)	*Thorichthys ellioti*	South-eastern, Mexico	Pinacho-Pinacho et al. [35]	KY077085

* Numbers in parentheses are the number of sequences used for the phylogenetic analysis.

Sequences were aligned in Clustal as implemented in MEGA 7 [26], using default parameter values. Observed genetic *p-*distances (*p*) between haplotype and sample pairs were calculated in MEGA 7. Phylogenetic relationships were inferred via maximum likelihood analyses using IQ-TREE [33], as implemented in the online W-IQ-TREE (http://iqtree.cibiv.univie.ac.at; [40]). The IQ-TREE software was also used to select the model of nucleotide substitution (TPM3 + G4). Support for clades found in the most likely tree was calculated via the SH-aLRT test [21] and with 1000 ultrafast bootstrap pseudo-replications (BL) also with IQ-TREE.

## Results

Specimens of *H. holospinus* are recorded from a new host, *L. equulus* (family Leiognathidae) which was concurrently infected with other acanthocephalans, in a new locality off Quang Ninh at the Gulf of Tonkin near Halong Bay, where the type specimens were originally collected. *Heterosentis holospinus* is apparently wide spread in fish from at least two percid families in the Halong Bay area.

### New morphological observations of specimens from recent collection (Figs. 1–6)

**Figures 1–6:**
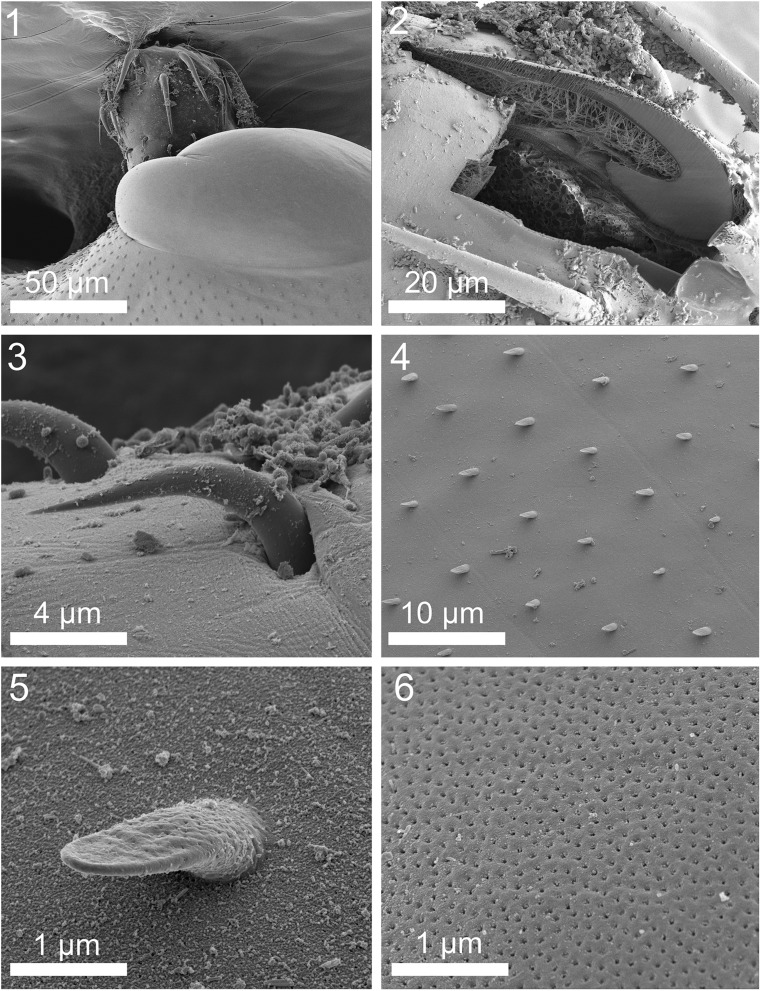
SEM of specimens of *Heterosentis holospinus* from *Leiognathus equulus* from Vietnam. (1) The anterior part of a worm showing the proboscis, the spineless anterior trunk cone, and the anterior part of the spiny trunk. (2) A longitudinal Gallium cut section of the anterior hook. Note the thickness of the hook layers and the relationship between the hook and the size and shape of the root. (3) Posterior hooks of the proboscis. (4) A general view of trunk spines and their pattern of distribution. (5) A high magnification of a typical duck-bill-shaped trunk spine showing a continuation of trunk micropores. (6) Micropores in a mid-section of the trunk.

Ten specimens of *H. holospinus* were collected from *Leiognathus equulus* Forsskål (Leiognathidae) in Quang Binh, Gulf of Tonkin on May 15, 2017 [22]. Six of these specimens were processed for microscopical studies. We provide morphological comparisons with the original description by Amin et al. [3]. The six specimens (one male and five gravid females) were similar to those described earlier [3], but with some disparities. Comparable and new descriptive features were noted in the SEM images, including the proboscis with apical and larger subapical hooks, unspined anterior trunk cone, and post-cone spined trunk (Fig. 1), one of 3–4 small spine-like hooks in a row (Fig. 2), a field of trunk spines (Fig. 3), a single magnified trunk spine exhibiting its unusual duck-billed shape and its innervation with micropores like those in the adjacent body wall (Figs. 4 and 5), and the posterior end of a female specimen showing the posterior-most distribution of trunk spines (Fig. 6). We also observed the characteristic receptacle shape, the plump longer lemnisci, and trunk shape, and size. The male reproductive structures, especially the shape of the thick sperm ducts, the rounded bursa, and the shape, and position of the testes and cement glands were comparable to those noted in the original description. The female reproductive system especially the complex vagina, the prominent and widening uterus, the uterine bell with many cells, and the eggs were also very similar. The nucleated pouch at the posterior end of the receptacle was, however, not consistently prominent and the trunk spines in the microscope specimens were faint and less readily distinct.

### Energy dispersive X-ray analysis (EDXA)

**Figure 7:**
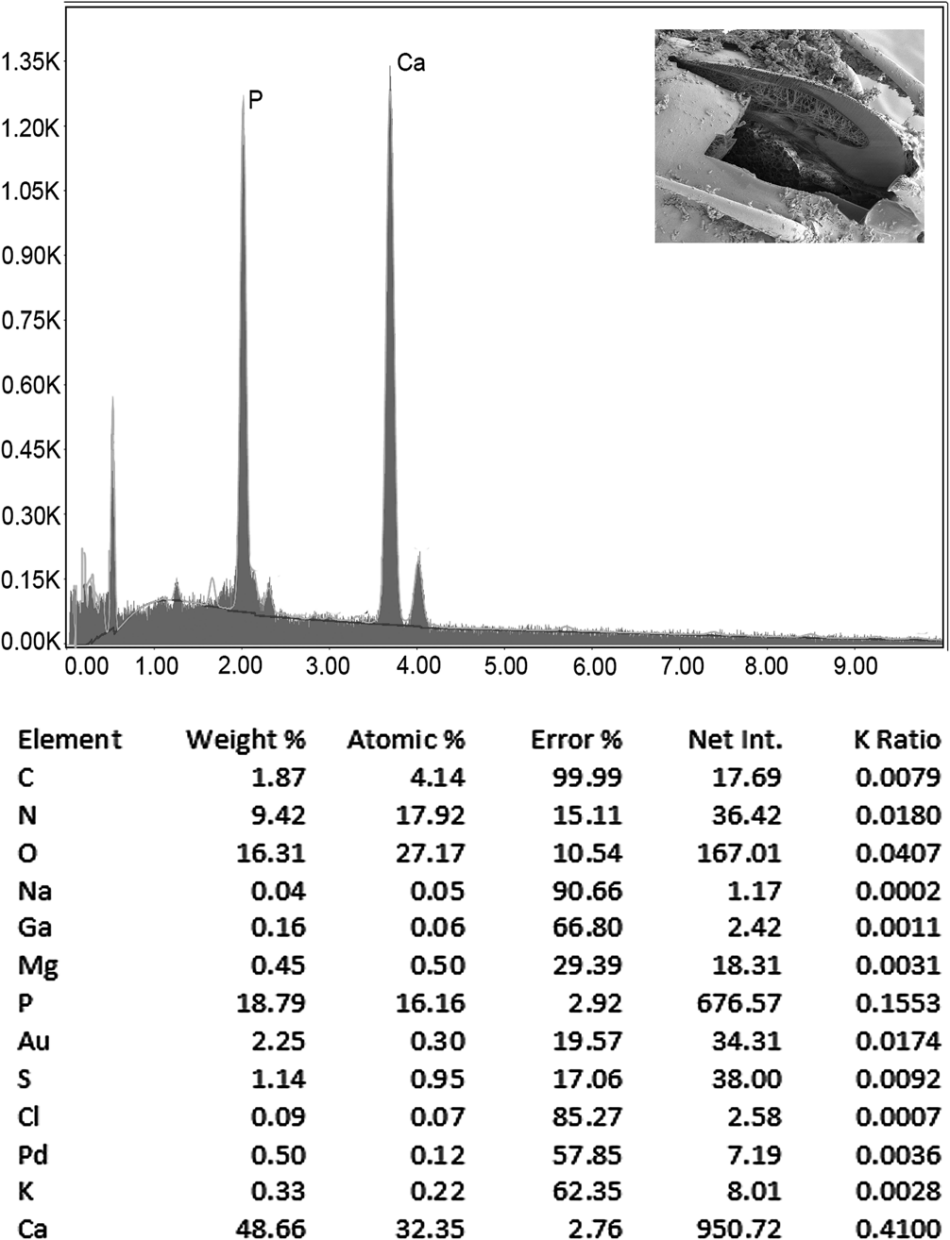
Energy dispersive X-ray spectrum of the basal arch of a Gallium cut hook of a *Heterosentis holospinus* specimen showing high levels of calcium and phosphorus similar to those of the hook base (Table 2). The X-ray data are the elemental analysis of the hook arch (see bolded figures in Table 2). Insert: SEM of a longitudinal Gallium cut hook.

**Figure 8:**
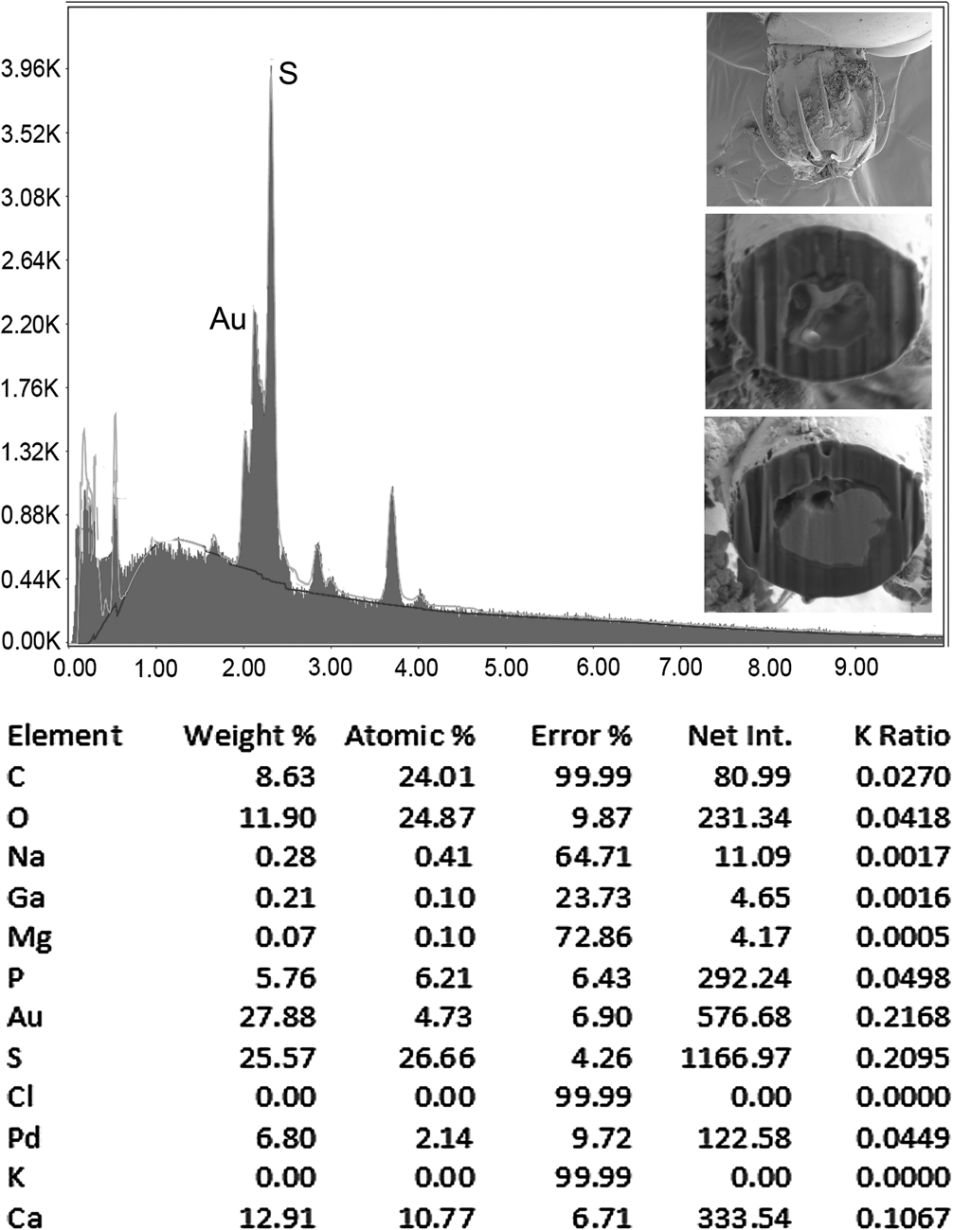
Energy Dispersive X-Ray spectrum of a Gallium cut hook tip of a *Heterosentis holospinus* specimen showing high levels of sulfur similar to those of the hook edge at mid-cut (Table 2). The X-ray data are the elemental analysis of the hook tip (see bolded figures in Table 2). Insert: SEM of a whole proboscis and cross sections near the hook tip of Gallium cut hooks showing the thick, high-sulfur hook edge.

Figures 7 and 8, and Table 2 demonstrate that different parts of the hook vary in the composition and distribution of metals with the basal hook arch having a considerably higher level of calcium and phosphorus but lower level of sulfur than the hook tip and edge.

**Table 2:** Chemical composition of Gallium (LMIS) cut hooks of *Heterosentis holospinus*.*

	Cross section cuts			Longitudinal cuts**	
	Tip cuts	Mid cut			
Element	Edge***	Edge	Center	Arch***	Base
Sodium (Na)	**0.28**	0.13	0.43	**0.04**	0.14
Magnesium (Mg)	**0.07**	0.21	0.77	**0.45**	0.07
Phosphorous (P)	**5.76**	10.30	16.92	**18.79**	20.76
Sulfur (S)	**25.57**	28.15	4.41	**1.14**	1.06
Potassium (K)	**0.00**	0.00	0.05	**0.33**	0.48
Calcium (Ca)	**12.91**	18.70	40.41	**48.66**	60.53

* Listed in WT%. Common protoplasm (C, O, N) elements and processing elements (Au, Pd, Ga) are omitted from the table.

** We also checked the longitudinal cuts for chlorine (Cl). The worm had low levels (0.11 and 0.09) of the ion which are NOT reliable.

*** Bolded figures are used to generate the corresponding spectra (Figs. 7 and 8).

### Molecular results

**Figure 9:**
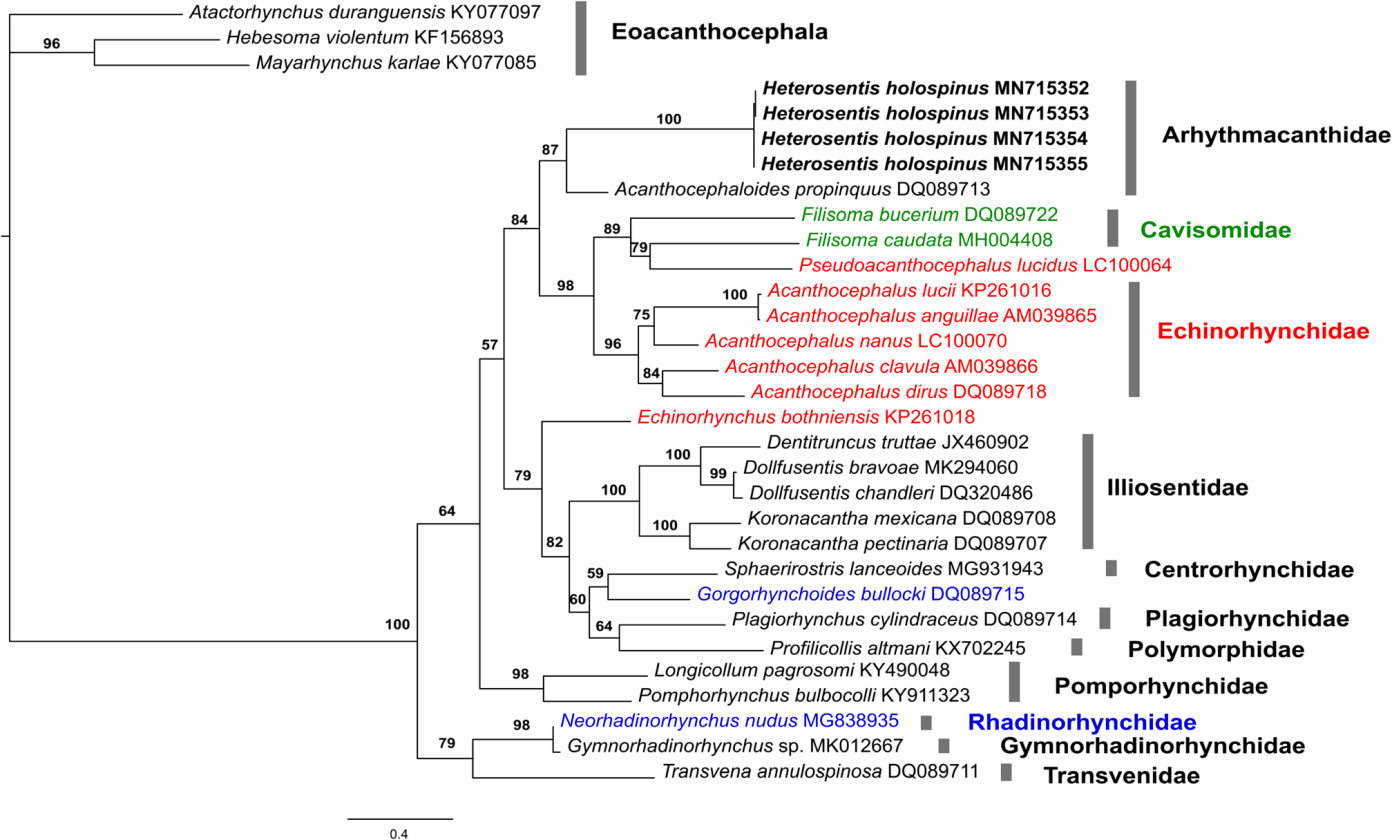
Genealogical relationships of haplotypes of the *cox*1 gene of specimens of *Heterosentis holospinus* and specimens of Class Palaeacanthocephala recovered in a maximum likelihood analysis (*L*_*n*_ = −11,079.294). Support values, only given for species and multispecies clades, correspond to SH-aLRT test and ultrabootstrap proportions. The number below the internal nodes shows ML bootstrap support values. The clades with different colors, Echinorhynchidae (red), Cavisomidae (green), and Rhadinorhynchidae (blue) indicate a paraphyletic arrangement. GenBank accession numbers are also given at the terminal labels and bolded names were generated in this study.

The phylogenetic tree of the Class Palaeacanthocephala (Fig. 9) is subdivided into eleven orders and three individuals belong to the Class Eoacanthocephala as outgroup. Within the order Arhythmacanthidae Yamaguti, 1935 and among the four sequences of *H. holospinus*, two distinct haplotypes were found (haplotype I [XX14–XX18]; haplotype II [XX02–XX04]), that showed a low level of genetic variation (0.5%). In the genealogical analysis (Fig. 9), these haplotypes form a strongly supported (BL = 100) monophyletic group, which is sister to *Acanthocephaloides propinquus* (Dujardin, 1845) Meyer, 1932 in a moderately supported clade (BL = 87) corresponding to Arhythmacanthidae. Sequences of *H. holospinus* and *A. propinquus* and order Arhythmacanthidae and Eoacanthocephalans sequences differ on average by 28% and 45%, respectively.

Regarding other palaeacanthocephalan families, two are not monophyletic. One of these is Echinorhynchidae as per analyzed species of the family. However, *Echinorhynchus bothniensis* Zdzitowiecki et Valtonen, 1987 forms a paraphyletic group to Cavisomidae, as *Pseudoacanthocephalus* Petrochenko, 1956 falls within *Filisoma* Van Cleave, 1928. Meanwhile, *E. bothniensis* constitutes the second lineage of echinorhynchids that is a highly divergent lineage within the radiation of Palaeacanthocephala. Also, *E. bothniensis* falls in a moderately supported clade (BL = 79) with Illiosentidae, Centrorhynchidae, Plagiorhynchidae, and Polymorphidae. The second family that is not monophyletic is Rhadinorhynchidae. *Neorhadinorhynchus nudus* (Harada, 1938) Yamaguti, 1939 forms a part of the main clade (BL = 79) of Palaeacanthocephala together with (BL = 98) Gymnorhadinorhynchidae, Transvenidae, which is sister (BL = 79) to the clade *N. nudus*-Gymnorhadinorhynchidae, and the family Pomphorhynchidae (BL = 98). Meanwhile, *Gorgorhynchoides bullocki* Cable and Mafarachisi, 1970 appear as sisters, in a weakly supported (BL = 59) relationship, to Centrorhynchidae. This latter clade is sister (BL = 60) to the clade (BL = 82) formed by Plagiorhynchidae and Polymorphidae. Finally, Illiosentidae (BL = 100) is sister to all other palaeacanthocephalans.

## Discussion

### Morphology

A number features observed in this collection provided an enhanced description of *H. holospinus* that was not available in previous material and confirmed its morphological identity from a second host species, *L. Equulus*, in a second family (Leiognathidae) in a new locality. New details of the anterior trunk cone, proboscis hooks, micropores, wholly spined trunk, duck-bill-like spines with micropores, and micropore distribution, increase our understanding of the anatomy of this species. New information on the unique chemical composition of its hooks added new elements to its description supporting its unique “personality.”

### Micropores

The electron dense micropores present throughout epidermal surface of the trunk of *H. holospinus* have not been discussed in the two other descriptions of the species [3, 22]. The micropores of *H. holospinus*, like those reported from other species of the Acanthocephala, are associated with internal crypts and vary in diameter and distribution in different trunk regions corresponding to differential absorption of nutrients. We have reported micropores in a large number of acanthocephalan species [23] and in a few more since, and demonstrated the tunneling from the cuticular surface into the internal crypts by TEM. Amin et al. [2] gave a summary of the structural–functional relationship of the micropores in various acanthocephalan species, including *Rhadinorhynchus ornatus* Van Cleave, 1918, *Polymorphus minutus* (Goeze, 1782) Lühe, 1911, *Moniliformis moniliformis* (Bremser, 1811) Travassos (1915), *Macracanthorhynchus hirudinaceus* (Pallas, 1781) Travassos (1916, 1917), and *Sclerocollum rubrimaris* Schmidt and Paperna, 1978. Byram and Fisher [13] and Wright and Lumsden [44] reported that the peripheral canals of the micropores are continuous with canalicular crypts. These crypts appear to “constitute a huge increase in external surface area… implicated in nutrient up take.” Whitfield [43] estimated a 44-fold increase at a surface density of 15 invaginations per 1 μm^2^ of *Moniliformis moniliformis* (Bremser, 1811) Travassos, 1915 tegumental surface. The micropores and the peripheral canal connections to the canaliculi of the inner layer of the tegument of *Corynosoma strumosum* (Rudolphi, 1802) Lühe, 1904 from the Caspian seal *Pusa caspica* (Gmelin) in the Caspian Sea were demonstrated by transmission electron micrographs in Amin et al. [4]. All the micropores that have been studied have the same basic plan; presumably because they have the same functions and similar variations, due to differential absorption occurring across the species that have been studied.

### Energy dispersive X-ray analysis (EDXA)

Results of the X-ray scans of the gallium cut hooks (dual beam SEM) of *H. holospinus* show differential composition and distribution of metals in different hook parts, with the calcium and phosphorus being considerably higher at the basal arch of hooks where tension and strength are paramount compared to the hook tip and edge where the level of sulfur was considerably higher (Table 2, Figs. 7 and 8). The chemical elements present in the hooks are typical for acanthocephalans [1, 5, 6, 22]. Note the thin outer layer (Fig. 2) of the hook which relates to the sulfur (*S*) content (Table 2) in the hook of *H. holospinus,* which is less than in other acanthocephalans [1, 6]. The high sulfur content shows up in the outer edge of X-ray scans of hooks (Table 2, Amin et al. [6]). The hook center in mid cuts has a completely different chemical profile than the cortical layer (Table 2). X-ray scans (EDXA) provide insight into the hardened components, e.g., calcium and phosphorus, of acanthocephalan hooks. The EDXA appears to be species-specific, as in finger prints, and is shown to have significant diagnostic value in acanthocephalan systematics, e.g. *Moniliformis cryptosaudi* Amin, Heckmann, Sharifdini, & Albayati, 2019 was erected based primarily on its EDXA pattern [8].

### Molecular discussion

The molecular analysis showed that *H. holospinus*, parasite of *L. equulus* from Vietnam, exhibits a low level of genetic variation; recovered haplotypes differ on average by 0.5%. At the same time, *H. holospinus* is highly divergent (28%) from *A. propinquus*, the other member of Arhythmacanthidae included in the analysis, and this species, in the present analysis, appears as its sister species. The limited size of the analyzed sample (four specimens) may provide a biased picture on the genetic diversity of the species. Nevertheless, this may not be the only cause of the low level of genetic diversity observed. Similar results were found for the genus *Profilicollis* Meyer, 1931 [30, 38]. The results of the present study should be seen as the starting point for clarifying the level and patterns of genetic variation in *H. holospinus*.

Various studies have suggested that the classification of acanthocephalans based only on morphological characters is unstable due to the existence of a conservative and simple morphology [12, 19, 34, 36]. Our findings, in accordance with those of previous studies [17, 36, 41] substantiate that the classification of Palaeacanthocephala needs adjustments. We found that two families, Echinorhynchidae and Rhadinorhynchidae, are not monophyletic. This finding is somewhat not surprising as acanthocephalan classification is mostly based on morphologic characters that may be prone to convergence. Additional analyses, including denser taxonomic and character sampling is much needed in order to obtain a robust phylogeny that would support a more stable classification and be the backbone for evolutionary and ecology studies.
